# Diagnostic antenatal d’une omphalocele au premier trimester

**DOI:** 10.11604/pamj.2016.25.187.10901

**Published:** 2016-11-24

**Authors:** Amira Ayachi, Mechaal Mourali

**Affiliations:** 1 Service de Gynécologie et Obstétrique, CHU Bougatfa, Bizerte Université Tunis El Manar, Tunisie

**Keywords:** Omphalocèle, échographie, caryotype, Omphalocele, ultrasound, caryotype

## Image en médecine

Nous illustrons à travers ce cas l'utilité du dépistage précoce des malformations fœtales au sein d'une unité de diagnostic anténatal, à travers les images d'une omphalocèle diagnostiquée au premier trimestre de la grossesse. Il s'agit d'une patiente âgée de 42 ans, G2P1, ayant fait une première échographie à 12SA+4 jrs montrant une omphalocèle (A,B,C,D). Les informations sur les différentes investigations ont été expliquées à la patiente, qui a décidé de poursuivre la grossesse. Une amniocentèse a été réalisée à 16 SA en vue d'un caryotype. La patiente reconsulte à 19 SA pour réévaluation échographique et interprétation des résultats du caryotype. L'échographie a montré une omphalocèle mesurant 4cm de diamètre, avec un collet à 14,3 mm, contenant le foie et l'estomac (E,F). Le résultat du caryotype révélait une trisomie 18. Une interruption médicalisée de la grossesse a été demandée par la patiente.

**Figure 1:**
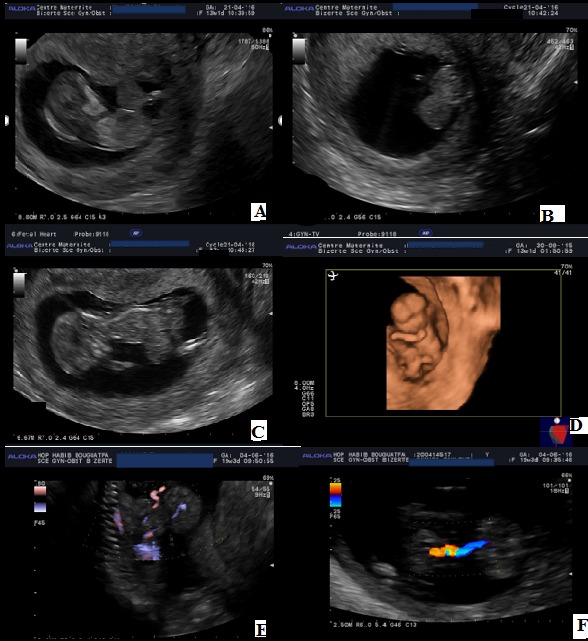
A, C) coupe LCC montrant l’omphalocèle; B) coupe transversale mettant en évidence le sac tout autour du défect; D) échographie en mode 3D montrant l’omphalocèle; E) contenu hépatique de l’omphalocèle; F) axe mésentérique

